# Anterolateral thigh flaps in closing large abdominal wall defect after the resection of mucinous adenocarcinoma: a case report

**DOI:** 10.1186/s12893-022-01550-x

**Published:** 2022-03-18

**Authors:** Weijia Huang, Hanpeng Lu, Yu-Xiao Zhang, Yinghan Song

**Affiliations:** 1 Department of Day Surgery Center, West China Hospital, Sichuan University, No. 37 Guoxue Alley, 610041 Chengdu, China; 2 Lung Cancer Center, West China Hospital, Sichuan University, Chengdu, China; 3 West China School of Medicine/West China Hospital, Sichuan University, Chengdu, China

**Keywords:** Abdominal wall defect, Mucinous adenocarcinoma, Abdominoplasty, Herniorrhaphy, Case report

## Abstract

**Background:**

It is a big challenge to repair a large abdominal wall defect after tumor resection, and en bloc resection with vascularized tissue reconstruction might be an alternative to achieve an improved survival for abdominal wall tumors.

**Case presentation:**

A 45-year-old woman presented with a 1-year history of persistent abdominal pain of the right lower quadrant and a mass with dermal ulceration. An enhanced computed tomography scan and biopsy of the mass were performed to achieve the definite diagnosis of abdominal mucinous adenocarcinoma. After four courses of “FOLFOX” chemotherapy, the tumor grew to 6 × 5 cm during preoperative examination. Thereafter, we removed the tumor and involved tissues and organs and repaired the sizeable abdominal wall defect used by biological meshes and vascularized anterolateral thigh flaps. The patient suffered green drainage of 450 ml in the abdominal cavity and intestinal anastomotic fistula, for which she readmitted and recovered afterward.

**Conclusions:**

Biological mesh combined with vascularized anterolateral thigh flaps could effectively repair the large abdominal wall defect and restore the biological function of the abdominal wall.

## Background

The reconstruction of the abdominal wall would protect the integrity of abdominal structures, keep the intraabdominal pressure, and retain the abdominal function. A sizeable abdominal defect is usually caused by tumor resection, trauma, burn, and so on [1, 2]. Mesh implanting and component separation technique(CST) are commonly available for the abdominal wall defect with a smaller size(< 40 cm^2^), while both of which might lead to tissue necrosis and abdominal compartment syndrome for the abdominal wall defects with a larger size(> 40 cm^2^). Therefore, vascularized tissues would be employed [3] with contamination resistance and angiogenesis promotion [4, 5].

## Case presentation

A 45-year-old woman presented a one-year history of persistent abdominal pain of the right lower quadrant and a mass with dermal ulceration. One year before admission, the pain occurred without obvious triggers, and the laparoscopy showed a cystic mass in the right lower quadrant of the abdominal cavity. Then, the patient was prescribed oral Chinese herbal medicine without others being performed at the local hospital. During the treatment, the pain recurred and became even worse with the formation of dermal ulceration, and the mass developed even larger after two times attempts of drainage of the purulent group with nothing draining out. After four courses of regular “FOLFOX” chemotherapy, the abdominal pain persisted in the right lower quadrant, and swelling pain occurred on the right back with a mass protruding from the epidermis. She had a history of appendectomy, colonic polypectomy, and left ovarian cyst resection.

**Fig. 1:**
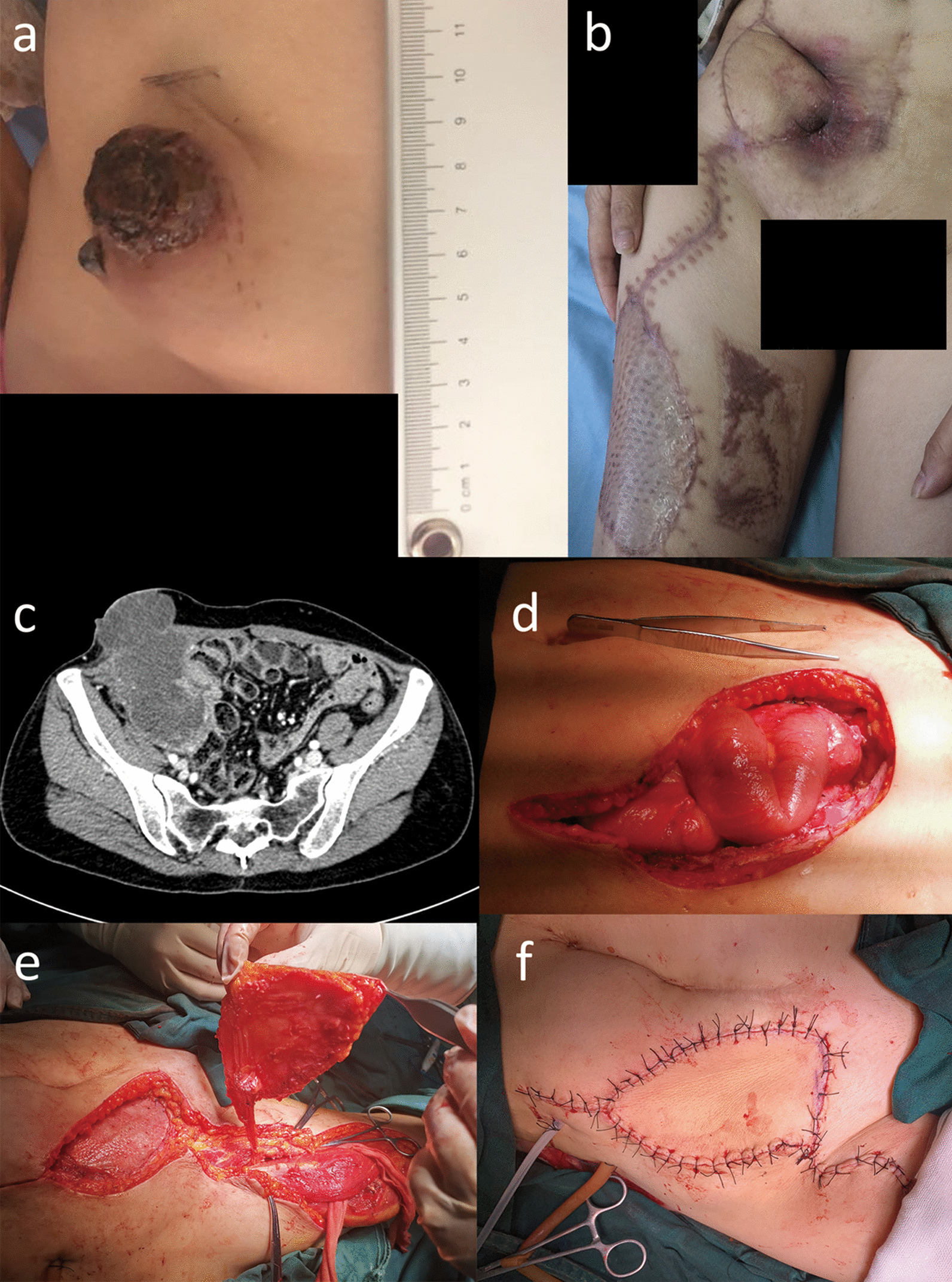
The preoperative appearance, intraoperative process, and postoperative appearance when follow-up. **a** the mucinous adenocarcinoma was examined in vivo preoperatively; **b** the fistula was closed, and the patient received the third course of chemotherapy five months postoperatively; **c** the tumor displayed on preoperative contrast-enhanced computed tomography; **d** the large abdominal wall defect (approximately 87 cm^2^) after resection of mucinous adenocarcinoma on the abdominal wall; **e** placement of a drainage tube in the abdominal cavity, mesh implanting (approximately 170 cm^2^, Cook Group Inc., U.S.) of the abdominal wall defect, and dissection of anterolateral vascularized thigh flaps from doner site; **f** placement of another drainage tube under the flap and suturing the skin

A rigid mass with a 6 × 5 cm size protruded from the skin without apparent tenderness and fluctuation(Fig. 1a), which lacked mobilization and could not be distinguished by the tissues around. The primary mass originated from the right part of the pelvis and invaded the uterus, bowels, right adnexa, and whole abdominal wall layers (Fig. 1c). Then it was diagnosed as abdominal low-grade mucinous adenocarcinoma(cT4N0M0 stage IIB, ypT4N0M0), proved by pathological puncture biopsy.

Subsequently, we resected the tumor and adjacent tissues and repaired the large abdominal wall defect(Fig. 1d). During surgery, the terminal ileum, ileocecal junction, and proximal ascending colon were found tightly adhered to the abdominal wall and the tumor. We resected the tumor and 15 cm of the ileum and conducted hand-sewn end-to-end anastomosis of the bowel, and the abdominal wall defect was approximately 87 cm^2^. Moreover, we also conducted enterolysis and right ureteral exploration intraoperatively.

After flushing the abdominal cavity thoroughly and placing a silicone drainage tube in the right iliac fossa, we performed a tension-free repair of abdominal wall defects by COOK biological patch(170 cm^2^, Cook Group Inc., U.S.) with continuous suture and 2 cm of the mesh overlapping on the edge of abdominal wall defect. Afterward, we consulted the plastic surgeons for the reconstruction and performed the anterolateral femoral free flap transfer nourished by the perforator branch vessels(Fig. 1e). Another silicone drainage tube was placed under the flap without suction, and gauze was placed above the flap for local compression hemostasis(Fig. 1f).

The patient got antibacterial and nutrition support treatment postoperatively. On the 16th day postoperatively, 450 ml of drainage in dark green was found in the abdominal cavity and 50 ml of drainage in brown subcutaneously. After symptomatic and antibacterial treatment, the patient went well and was discharged 37 days postoperatively. She was readmitted 40 days postoperatively due to an intestinal anastomotic fistula, and it was controlled after symptomatic therapy in the second admission. The patient was discharged without discomfort and followed up actively after discharge, and she had been followed up for five months since the operation(Fig. 1b).

## Discussion and conclusions

Nowadays, several studies have indicated that using anterolateral thigh flaps in free flap transfer was practicable, which could provide extensive coverage and vascularized fascia structures and minimize the influence on abdominal wall mechanics [6–10]. Song also used tensor fascia lata (TFL) as the donor site, which would cover at most 40 × 25 cm^2^ [11], while it was indicated that the recurrence rate of hernia was up to 40% when using TFL alone [12]. Due to the challenges in dissecting intramuscular perforators, implementing flaps with femoris lateralis and perforators would be an alternative to boost success [13].

It remains controversial whether synthetic or biological meshes are necessary to reconstruct abdominal wall defects. In this case, we closed the abdominal wall used by biological mesh before the cover of free flaps, which was available to enhance the reconstruction function and counteract the tension on the suture [14]. For fear of the inflammatory response due to the meshes, Sugarbaker repaired the defect by contralateral rectus abdominis muscle instead to facilitate secondary surgery among people who recurred after abdominal or pelvic cancer resection required further treatments [15]. Considering biological meshes are contamination resistant, it was available to use biological meshes in potentially infected wounds [11, 16–19]. Sun compared the safety and efficacy of biological meshes and polypropylene meshes in hernia repair, and they found that biological meshes would lead to less pain compared with polypropylene meshes (P < 0.001) [20]. In contrast, Warren showed that permanent synthetic mesh might perform better in safety, bacteria control, and low recurrence than biological meshes and bioabsorbable meshes [21].

Moreover, a late anastomotic leakage happened in this patient, a potential and unforeseen adverse event. It might be associated with anastomotic techniques (stapled versus hand-sewn anastomoses) and surgeon’s specialism, while no conclusions had been drawn to reveal the specific risk factors [22, 23]. Furthermore, even though the leakage happened around the wound, the flap transfer still worked in the contaminated region.

In conclusion, the anterolateral thigh flap transfer is safe and effective for patients with large abdominal defects after resection of abdominal wall malignancy. At the same time, the safety and efficacy of anterolateral thigh flap transfer remained to be further studied in future research, including surgical procedures and selection of meshes.

## Data Availability

All data generated or analyzed during this study are included in this published article.

## Abbreviations

CST: Component separation technique
TFL: tensor fascia lata
